# Auditory-Induced Emotion Mediates Perceptual Categorization of Everyday Sounds

**DOI:** 10.3389/fpsyg.2016.01565

**Published:** 2016-10-13

**Authors:** Penny Bergman, Daniel Västfjäll, Ana Tajadura-Jiménez, Erkin Asutay

**Affiliations:** ^1^SP, Sveriges Tekniska ForskningsinstitutBorås, Sweden; ^2^Department of Behavioral Sciences and Learning, Linköping UniversitySweden; ^3^Department of Psychology, Universidad Loyola AndalucíaSeville, Spain

**Keywords:** auditory-induced emotion, auditory perception, categorization, everyday sounds, perceptual decisions

## Abstract

Research has shown that emotion categorization plays an important role in perception and categorization in the visual domain. In the present paper, we investigated the role of auditory-induced emotions for auditory perception. We further investigated whether the emotional responses mediate other perceptual judgments of sounds. In an experiment, participants either rated general dissimilarities between sounds or dissimilarities of specific aspects of sounds. The results showed that the general perceptual salience map could be explained by both the emotional responses to, and perceptual aspects of, the sounds. Importantly, the perceptual aspects were mediated by emotional responses. Together these results show that emotions are an integral part of auditory perception that is used as the intuitive basis for categorizing everyday sounds.

## Introduction

Emotions are central to our perception of the environment surrounding us (Berlyne, 1971). Yet, we know relatively little about how and when emotions are elicited by environmental factors and how they affect categorization and judgments about the environment. Building on recent research showing that sounds occurring in an environment often induce emotions (Västfjäll et al., 2003; Juslin and Västfjäll, 2008; Tajadura-Jiménez et al., 2010a,b) the present study investigated the role of emotions in the categorization and discrimination of sounds. It further investigated if perceptual decisions about sounds are primarily based on the emotions induced by the sounds (auditory-induced emotions), rather than the perceived physical properties of the sounds.

Our perception of the environment is shaped by how we categorize the objects in it. A broad distinction can be made between similarity and theory approaches to categorization. Similarity represents the individuals' understanding of the natural correlations of the physical stimuli, i.e., how similar the stimuli are in terms of their physical aspects. Theory-categorization includes several different aspects, which may change depending on context but are mainly used from a strict cognitive perspective. Similarity judgments are based on the underlying idea or deeper structure of the objects including meaning and functionality (Niedenthal et al., 1999). Emotion-categorization, on the other hand, is based on the notion that objects that elicit the same kind of emotions are grouped together (Niedenthal et al., 1999). Emotional responses as a basis for categorization may be distinguished from both similarity and theory categorization, as it is not the external feature of the stimulus but the listeners' internal responses that is used for categorization. These emotional responses are prioritized due to its direct relevance for our well-being and survival (Brosch et al., 2009).

Auditory stimuli tend to evoke relatively strong emotional reactions (Bradley and Lang, 2000). It is therefore likely that participants also use their emotional responses as decision criterion when categorizing auditory signals. Auditory-induced emotion as basis for categorization is a neglected topic in the research on sound perception (with the exception of musically induced emotion; see Juslin and Västfjäll, 2008). Non-musical sound perception, which is the focus of the present research, and categorization have primarily been studied from the view-point of physical similarity with the aim to find acoustic correlates that explain perception (Caclin et al., 2005). Only recently, theory-categorization has started to receive some attention in the field of non-musical sound perception. Gygi et al. (2007) examined categorization of environmental sounds and showed that apart from the physical similarity, the source to the sound (included in “theory categorization”) was also an important ground for categorization. Dubois (2000) showed that participants tend to categorize sounds rather by the event causing the sound (i.e., theory-categorization) than similarity and only when identification of the sound fails the categorization is conducted in terms of physical similarity. A recent study showed a differentiation between “living” vs. “nonliving” sounds: sounds produced by nonliving sources were categorized by the physical similarity whereas sounds from living sources were categorized and structured by the symbolism and theory behind (Giordano et al., 2010). The influence of auditory-induced emotions for categorization is however not yet examined. There are good reasons to think that physical characteristics of a sound may differ, yet the induced emotions are similar. For instance, research has shown that low-pitched loud screams may induce a similar reaction (i.e., fear) such as high-pitched and less loud hissing sounds (i.e., rattlesnake).

Dissimilarity ratings are a tool to distinguish the prevalent or dominant perceptual feature of a stimulus. The dissimilarity ratings are then analyzed by Multi-Dimensional Scaling (MDS) methods to produce a so called “perceptual map.” The perceptual map is interpreted as reflecting the perceptual salience of the parameters (Miller and Carterette, 1975).

We hypothesized that the perceptual map of a given set of sounds could be explained by the emotion- and theory-categorization rather than by similarity. Previous research in the visual domain suggests that the emotional response to an object aids the identification and perception of the object (Russell, 2003; Adolphs, 2004; Zeelenberg et al., 2006). Barrett and Bar (2009) showed that the emotional response assists in seeing an object as what it is from the very moment the visual stimulation begins. They argued that when the brain detects visual sensations from the eyes it uses not only previously encountered pattern of form and theory, but also the affective representation. We hypothesized that a similar form of processing would occur in the auditory domain. Thus, the perceptual ratings of a sound should be mediated by the emotional reactions to that sound.

### The present study

The present study investigated whether categorization of environmental sounds may be accounted for by participants' emotional reactions to the same sounds, characterized in terms of valence and arousal. We further explored if perceptual characteristics are related to the main dimensions of the Perceptual map. If that was the case we expected these to be mediated by emotional reactions to the sounds. The hypothesis was investigated by means of several dissimilarity ratings and a set of mediation analyses. Having participants judging the general dissimilarity of sounds allows producing a representation of the most salient parameters in the stimuli set. Previous studies have shown that the dimensions of the perceptual map derived by a multidimensional scaling technique are explained partly by perceptual characteristics in the sounds (Gygi et al., 2004, 2007; Reddy et al., 2009).

## Methods

### Participants

Twelve participants participated in two experiments, 8 male and 4 female. The average age was 29 years (*SD* = 4.6). They were paid for their participation and gave their informed consent prior to the inclusion in the study. The current study was conducted under approval of the local ethics committee.

### Design

Twelve short sounds from the International Affective Digital Sounds (IADS; Bradley and Lang, 1999) were used. The sounds were chosen as to have three levels of affective content in terms of valence (pleasant, neutral, and unpleasant). Each affective group consisted of four sounds from four different categories: human sounds, animal sounds, mechanical sounds, and environment sounds. The sounds were equally spread between “living” and “non-living” sources. For a list of the sounds used, see Table 1. All the 12 sounds chosen were common everyday sounds that the participants should have experienced before to avoid a novelty effect. The sounds were presented in a half matrix design, thus resulting in 66 pairs of sounds. The sound pairs were randomized in order and presented counterbalanced both in order within each pair and order of the 66 pairs, thus resulting in four different orders presented to the participants.

**Table 1:** **The results of the affective ratings of the 12 sounds**.

**Description**	**IADS no**.	**Category**	**Valence^a^**	**Arousal^a^**	**Valence^b^**	**Arousal^b^**
**LOW VALENCE**						
Baby cry	261	Human	2.84	6.49	2.67	6.50
Dog growl	106	Animal	2.73	7.77	2.83	7.75
Car wreck	424	Environ.	1.95	7.82	2.17	7.92
Jackhammer	380	Mechanical	3.66	5.6	2.67	6.50
**NEUTRAL VALENCE**						
Yawn	262	Human	5.32	2.01	5.67	2.17
Chickens	132	Animal	5.55	3.93	5.75	4.92
Thunderstorm	602	Environ.	5.29	3.85	6.08	3.58
Clocktick	708	Mechanical	4.38	4.56	4.42	3.50
**HIGH VALENCE**						
Baby laugh	110	Human	7.92	6.04	7.92	6.33
Cardinal	151	Animal	7.35	2.73	7.42	3.50
Roller coaster	360	Environ.	6.9	7.36	6.67	6.50
Beer	721	Mechanical	7.13	4.43	7.33	4.17

In the table divided by the three valence categories: low valence, neutral valence, and high valence. The 4th and the 5th column represent the ratings from Bradley and Lang (1999) and the 6th and 7th column represent the ratings in present study.

a Valence and Arousal ratings according to the IADS (Bradley and Lang, 1999).

b Valence and Arousal ratings from this experiment.

### Procedure

The participants were individually tested in a sound-attenuated room at two occasions. At the first occasion the participants were instructed in how to use the dissimilarity rating scale and asked to rate the dissimilarity of pair-wise presented sounds, with no further instructions on what to attend to. After the dissimilarity ratings were completed the participants rated each sound individually on scales of valence and arousal using the 9-point Self-Assessment Manikin scale (Bradley and Lang, 1999).

At the second occasion the participants did focused dissimilarity ratings for the pairwise presented sounds on seven scales. The first two scales asked for how dissimilar the sounds were in terms of how pleasant and how activating they were. The remaining five scales asked for aspects concerning their perception of physical similarity in the sounds regarding, (1) Complexity, (2) Loudness, (3) Sharpness, (4) Speed of attack, and (5) Decay time. While not exhaustive, these perceptual parameters have been shown to account for the main bulk of variance in emotional reactions to both meaningful and meaningless sounds (Västfjäll et al., 2003; Asutay et al., 2012; Västfjäll, 2013). On both occasions the participants had full control over the playback rate and, if needed, they could repeat the sounds within the present pair but not go back to the previous pairs. The participants were at both occasions urged to use the entire scale while rating the differences. To be able to determine what the underlying dimensions of differentiation between stimuli are, as well as how many factors that are taken into account, different multidimensional scaling methods have been developed. Multidimensional scaling determines the psychological distance among different stimuli and in combination with other measures may determine what parameters that explain the psychological distance. This builds partly on the notion that we may only focus on a limited number of parameters while e.g., listening to complex stimuli and the aim is thus to establish the most perceptual salient parameters of the sounds (Miller and Carterette, 1975). Dissimilarity ratings may also be able to determine unknown relationships among the objects under study. Importantly, these relationships may not emerge using classical linear correlations.

Participants' dissimilarity ratings were therefore analyzed by use of the Individual Difference multidimensional Scaling (INDSCAL) technique developed by Carroll and Chang (1970). The general MDS model attempts to uncover latent psychological structure in a set of stimuli by maximizing the correspondence between some sort of paired similarity judgments and inter-stimulus distance in a multidimensional psychological space. The INDSCAL model assumes that all participants share the same psychological space but attends differently to the underlying psychological dimensions (Ashby et al., 1994). INDSCAL can provide a critical test of the selective attention hypothesis, because the dimension weights in the model represent the use of specific stimulus dimensions.

The complex nature of the physical environment, and several aspects that may be attained to it, make it difficult to identify a few factors that explain perception. Due to this the MDS solutions for complex stimuli often hold a higher stress level than those for simple stimuli (see Gygi et al., 2007) and may require a higher amount of dimensions to enable an understanding of the dimensions, i.e., to reduce the level of noise. More dimensions will however not alter which the explanatory factors are but make them easier to recognize.

## Results

The original raw data will be available at public data depository (https://www.researchgate.net/profile/Daniel_Vaestfjaell/contributions).

### Manipulation check

The manipulation check measure of the focused dissimilarity ratings for pleasantness and activation showed that both were explained by the rated emotional responses. The INDSCAL analysis of the pleasantness dissimilarity ratings rendered a three-dimensional model [stress = 0.227; Proportion of variance accounted for (RSQ) = 0.582]. The analysis of activation dissimilarity ratings rendered a three-dimensional model as well (stress = 0.222; RSQ = 0.582).

The results of the valence and arousal ratings are presented in Table 1. The valence and arousal ratings were ordered in hierarchical orders.

Linear regression analysis using the ratings of valence and arousal as independent variables and the MDS dimensions as dependent variables confirmed that valence and arousal separately accounted for two of the three dimensions in both the pleasant MDS as well as the activation MDS. *B* varied between 0.72 and 0.92 (all significant; *p* < 0.05).

### Perceptual map

**Figure 1:**
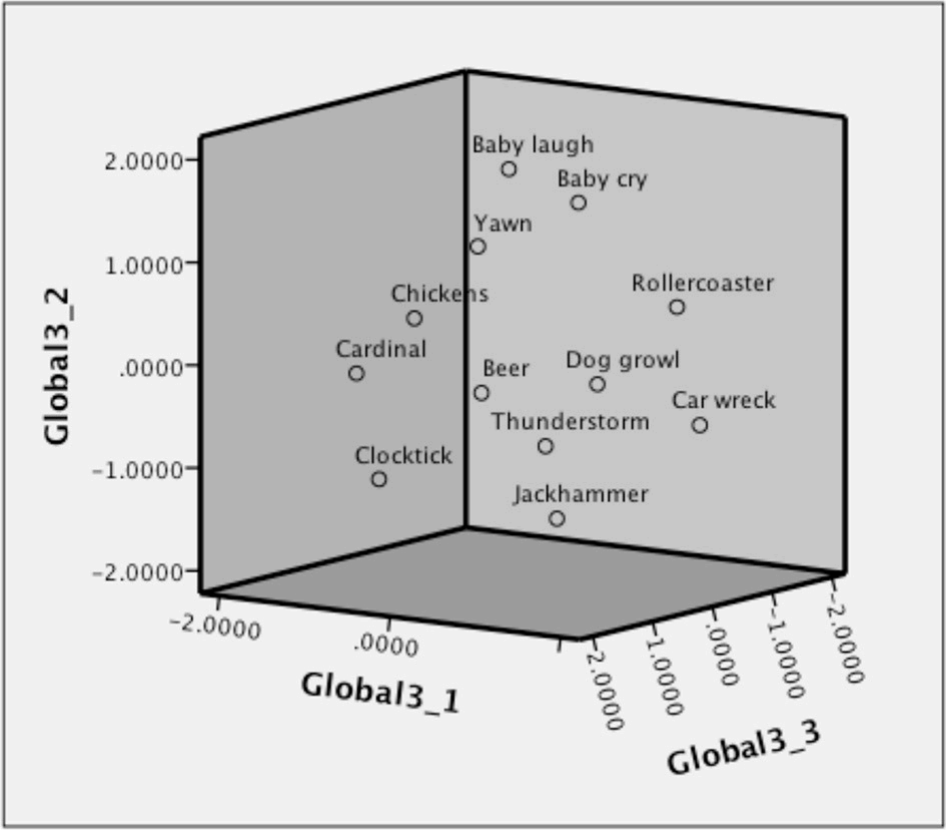
**3-Dimensional MDS solution for global dissimilarity**.

To test the main hypotheses, the global dissimilarity ratings were subjected to a MDS analysis, which yielded a 3-dimensional MDS models (see Figure 1 for the 3-dimensional global solution).

The main hypothesis is that if emotional reactions are driving the dissimilarity ratings, at least one of the dimensions of the MDS solution should be correlated with, and be explained the valence and/or arousal ratings. In support of this, we found that both the perceptual characteristics (in at least one dimension) as well as the valence and arousal ratings all proved to be able to explain the first dimension of the global ratings (the perceptual map; see Table 2). Thus, the first dimension of global judgments is, at least partly based on emotional reactions to the sounds. Interestingly, the decision criterions for the other two dimensions were not based on emotion categorization but rather on something that would be interpreted as a theory-approach to categorization. A dummy variables for living (human and animals) and nonliving (mechanical and environmental) sounds explained the second and third dimensions, *B* = 0.76, *p* > 0.01.

**Table 2:** **The results of the regression and mediation analyses**.

**Dep. Variable (Y)**	**Indep. variable (X)**	**Mediator (M)**	**Y = β + β_x_X**	**M = β + β_x_X**	**Y = β + β_x_X + β_M_M + ε**		**Z_Zobel_**
			**β_x_**	**β_x_**	**β_x_**	**β_M_**	
Perceptual map, 1 dim.	Complexity	Arousal ratings	0.734^**^	0.751^**^	0.105^n.s.^	0.839^**^	3.20^**^
	Loudness	Arousal ratings	−0.601^*^	−0.678^*^	0.039^n.s.^	0.943^**^	−2.68^**^
	Sharpness	Arousal ratings	−0.680^*^	−0.649^*^	−0.147^n.s.^	0.822^**^	−0.2.51^*^
	Attack	Arousal ratings	−0.811^**^	−0.888^**^	0.016^n.s.^	0.931^**^	−4.6^*^
	Decay	Arousal ratings	0.831^**^	0.867^**^	0.143^n.s.^	0.793^*^	4.36^**^
	Valence ratings	–	−0.638^*^	–	–	–	–
	Arousal ratings	–	0.917^**^	–	–	–	–

The table shows the effect on the first dimension of the perceptual map by the independent variables along with the results following the causal step method by Baron and Kenny (1986). The final column shows the z-scores from the modified Sobel test.

* Significant at 0.05-level.

** Significant at 0.01-level.

n.s. Non-significant.

### Mediation by emotion

Since both perceptual ratings and emotional ratings were associated with the primary dimension of perceptual space, we further tested if the association between perceptual decisions and the primary perceptual dimension of the perceptual map was mediated by emotions by conducting a set of mediation analyses. The mediation analyses assessed whether the perceptual characteristics affected the general multidimensional space and if that could be explained by the emotional reactions to the sounds. The first mediation analysis employed Baron and Kenny (1986) causal steps method.

For the mediation analysis the first dimension of the general MDS were used as the dependent variables (Y) and the ratings of perceptual characteristics were used as explanatory independent variables (X). As mediator (M) the ratings of valence and arousal from the individual ratings were used (see Table 2).

When perceptual characteristics and arousal were considered simultaneously the direct effect of perceptual characteristics on the dimensions was no longer significant in all of the five perceptual decision ratings. The effect of arousal ratings (the mediator) was, however, significant. This implies that all the perceptual characteristics were mediated by the arousal ratings. Valence did, however, not mediate in any case the perceptual ratings (see Table 2). To assess the extent to which arousal carried the effect of the independent variables on the dependent variable the modified Sobel test was conducted (MacKinnon et al., 2002).

Taken together, these results suggest that emotional arousal is a primary cue for categorization of everyday sounds.

## Discussion

This research sat out to show that emotion is an integral part of auditory perception—specifically that emotional responses to sound are used as a decision criterion when discriminating between sounds. The manipulation check showed that the multidimensional dissimilarity space for pleasantness and activation, were explained by ratings of experienced valence and arousal, respectively.

The results further showed that valence and arousal explained the first dimension of the perceptual map whereas theory-categorization accounted for the other two dimensions, i.e., listeners differentiated between living and nonliving sources in line with the results by Giordano et al. (2010). The results further showed that the physical similarity of the environmental sounds measured as complexity, loudness, sharpness, attack, and decay was mediated by arousal ratings thereby supporting the hypothesis that perceptual discrimination and grouping are mediated by the emotional reactions. Ratings of valence did, however, not mediate the perceptual ratings. This might be explained by the choices of sound stimuli and method: the sounds were chosen from three groups of valence (pleasant, neutral, and unpleasant), whereas the arousal ratings were less parametrically chosen and therefore the ratings were more evenly spread along the arousal scale than the valence scale. If more sounds had been added, a bigger spread within valence ratings could have been achieved and possibly resulted in an inclusion of valence as mediator for the perceptual ratings. The lack of results for valence ratings, and strong dominance of arousal, could possibly also point toward the notion that one of the primary functions of the auditory system is as an alarm system, where the main focus is to inform of potential threats (Juslin and Västfjäll, 2008). The sensitivity for arousal changes could therefore be stronger and more pronounced than sensitivity for changes in valence (this line of reasoning is consistent with arousal theories of esthetic perception; Berlyne, 1971). An alternative explanation could be a dominance of arousal-focused participants in line with the results of Barrett et al. (2004).

The findings from this study are important for how we understand auditory perception. The research on sound perception has so far primarily focused on the physical similarity of sounds. Emotional perception and emotional categorization have, relative to physical similarity, received little research attention in the field of sound perception. The results of this study add to this literature by showing that it is needed to consider emotional categorization in studies of environmental sounds. This research also contributes to emotion psychology, by showing that auditory-induced emotion is an intuitive basis for categorizing everyday sounds. This study thus extends previous research showing that emotions are used for categorizing visual stimuli (Barrett and Bar, 2009) to show that emotions are also used for categorizing auditory stimuli.

## Author contributions

Conceived and designed the experiments: PB, AT, EA, and DV. Performed the experiments: PB. Analyzed the data: PB. Wrote the paper: PB and DV.

## Funding

Research supported by the Swedish Research Council (VR). AT was supported by the MINECO Ramón y Cajal research contract RYC-2014-15421.

### Conflict of interest statement

The authors declare that the research was conducted in the absence of any commercial or financial relationships that could be construed as a potential conflict of interest.
